# A predictive study of glycaemic reversal in Chinese individuals with prediabetes based on machine learning: a 5-year cohort study

**DOI:** 10.3389/fendo.2026.1686082

**Published:** 2026-01-28

**Authors:** Changshun Yan, Su Hu, Hangyu Cao, Rui Xu, Guiqiu Cao, Genshan Ma

**Affiliations:** 1 State Key Laboratory of Pathogenesis, Prevention and Treatment of High Incidence Diseases in Central Asia, Xinjiang Medical University, Urumqi, Xinjiang, China; 2 Department of Cardiovascular Medicine, The Fifth Affiliated Hospital of Xinjiang Medical University, Urumqi, Xinjiang, China; 3 Department of Digital Science and Intelligent Software, South China University of Technology, Guangzhou, Guangdong, China; 4 Department of Cardiovascular Medicine, The Zhongda Hospital Southeast University, Nanjing, Jiangsu, China

**Keywords:** Chinese population, diabetes mellitus, machine learning, prediabetes, prediction models

## Abstract

**Objective:**

Diabetes mellitus (DM) poses a major global public health challenge. Prediabetes, a critical stage in the progression of DM, represents a pivotal window for intervention and prevention. This study aims to develop and validate a machine learning-based prediction model for glycemic reversal in Chinese individuals with prediabetes, with the goal of facilitating such reversal in this population.

**Methods:**

This study analyzed data of Chinese adults from the Dryad database, with a follow-up period from 2010 to 2016. LASSO regression was used to select variables. The selected variables were then used to construct models using random forest, gradient boosting decision tree, eXtreme gradient boosting, Naive Bayes, adaptive boosting, support vector machine (SVM), and Cox model. To assess the discriminative ability of each model, the area under the curve (AUC) was calculated for each. Predictive performance was evaluated by computing time-dependent AUC (t-AUC), accuracy, precision, recall, F1, and C-index. Shapley additive explanations (SHAP) analysis was applied to interpret the key variables identified by the optimal model, and Kaplan-Meier curves for key variables associated with glycemic improvement were plotted to explore differences between groups.

**Results:**

1792 adults with prediabetes were enrolled. During 5 years of follow-up, 942 achieved normoglycemia, yielding a reversal rate of 52.6%. After differential analysis and LASSO regression screening, 12 feature variables were finally determined for model construction. The 3-year, 4-year, and 5-year AUC values for the Cox model all exceeded 0.61. Six machine learning algorithms were employed to construct predictive models. The SVM demonstrated superior overall performance: it yielded a t-AUC of 0.711, accuracy of 0.652, precision of 0.620, recall of 0.661, F1 of 0.639, and a C-Index of 0.709, outperforming the other algorithms. SHAP analysis revealed that age, FPG, BMI, SBP, DBP, and triglycerides are key factors influencing normoglycemia reversal in individuals with prediabetes.

**Conclusion:**

We developed an SVM model to predict glycemic reversal in the prediabetic population in China, and identified key factors influencing glycemic improvement. This work provides a scientific basis for both this population and clinicians to implement early targeted interventions, thereby aiding in reducing the incidence of DM and alleviating the healthcare burden.

## Introduction

Type 2 diabetes mellitus (T2DM) has been designated by the World Health Organization (WHO) as one of the four priority non-communicable diseases. Between 1990 and 2019, the global prevalence of T2DM increased from 4,690.7 per 100,000 to 5,282.9 per 100,000, representing a 12.6% increase (1). Of note, prediabetes represents a high-risk state preceding T2DM, with diagnostic criteria defined in the American Diabetes Association (ADA) 2023 guidelines as fasting plasma glucose (FPG) 5.6 - 6.9 mmol/L and/or 2-hour plasma glucose (2-h PG) 7.8 - 11.0 mmol/L and/or glycated hemoglobin A1c (A1C) 5.7% - 6.4% (2). According to the International Diabetes Federation (IDF), approximately 352 million people worldwide are in a prediabetic state, a figure projected to increase to 587 million by 2045 (1, 3). Prediabetes is primarily characterized by mild insulin resistance (IR) and/or partial pancreatic β-cell dysfunction, and has been established as a high-risk factor for cardiovascular disease, chronic kidney disease, cancer, dementia, and all-cause mortality (4). Clinical evidence indicates that approximately 25% of individuals with prediabetes will progress to T2DM within 3–5 years, with the cumulative risk of T2DM development reaching up to 70% among all prediabetic individuals (5). Importantly, prediabetes is reversible. The IDPP-2 study demonstrated that 32.3% of prediabetic individuals can achieve normoglycemia following lifestyle interventions including dietary modification and exercise (6). The CRONICAS cohort study further confirmed that prediabetic individuals younger than 45 years with a body mass index (BMI) below 25 kg/m² have significantly higher rates of glucose normalization, with high-altitude environments and healthy lifestyles promoting glycemic reversal through enhanced insulin receptor sensitivity (7). Therefore, implementing targeted interventions for prediabetic individuals to improve glycemic control and facilitate their return to normoglycemia represents a critical strategy for controlling T2DM incidence and reducing the economic and service burden on public healthcare systems.

Machine learning algorithms have been widely applied in the field of diabetes mellitus (DM) and prediabetes, encompassing early diagnosis and risk prediction. Wang et al. (8) used data from a Japanese hospital database and only employed the Cox proportional hazards model to identify age, waist circumference, smoking history, fatty liver disease, and FPG as risk factors for incident prediabetes. The model achieved an area under the receiver operating characteristic curve (AUC) exceeding 0.8 in both the training set and validation set, demonstrating favorable predictive performance. However, this study failed to incorporate other models to validate the nonlinear relationships between variables, thereby limiting improvements in model interpretability and predictive efficacy. Choi et al. (9) took advantage of machine learning models’ ability to handle complex inter-variable relationships and developed artificial neural network (ANN) and support vector machine (SVM) models using data from the Korean National Health and Nutrition Examination Survey. These models were compared with a traditional logistic regression screening score model to predict the risk of incident prediabetes. Results showed that the SVM achieved the best performance, with an AUC of 0.731 which was higher than that of the ANN (0.729) and the traditional logistic regression model (0.712), along with superior accuracy. Nevertheless, the study used cross-sectional data to develop a static model, failing to capture the disease progression of prediabetes through long-term follow-up. Liu et al. (10) followed 6247 young Chinese men with normal FPG for a median of 5.8 years and developed prediabetes prediction models using random forest (RF), stochastic gradient boosting (SGB), eXtreme gradient boosting (XG Boost), and elastic net (EN). Among the predictors, FPG was the top factor associated with an increased risk of incident prediabetes, followed by body fat and creatinine. Compared with the multiple linear regression model, the machine learning models exhibited lower performance error and higher accuracy in outcome prediction. Although various models for predicting incident prediabetes have been developed using traditional models and machine learning algorithms, there remains a paucity of prediction models exploring glycemic reversal through long-term follow-up specifically for Chinese individuals with prediabetes.

Based on data from a 5-year cohort study conducted in China, we developed a predictive model for glycemic reversal in individuals with prediabetes by integrating machine learning algorithms with the Cox proportional hazards model. Interpretation of this model identified key factors influencing blood glucose dynamics, enabling clinicians and individuals with prediabetes to implement targeted interventions. Such interventions aim to facilitate the early reversal of blood glucose to normal levels, thereby improving population outcomes and alleviating the disease burden associated with progression to overt diabetes.

## Materials and methods

### Data source

The data used in this study were derived from the Dryad database (https://www.datadryad.org/stash). Co-founded by the Bioinformatics Institute of the University of Ottawa, Canada, and the Department of Ecology and Evolution of the National Academy of Sciences, USA, this database is dedicated to addressing issues related to the sharing and accessibility of scientific research data, providing open access to all researchers. The raw data were extracted by Chen et al. (11) from the electronic database of Rich Healthcare Group in China, encompassing medical records of individuals who underwent health examinations at 32 health check-up centers across 11 cities (Beijing, Shanghai, Guangzhou, Shenzhen, Nanjing, Suzhou, Changzhou, Chengdu, Hefei, Wuhan, and Nantong) between 2010 and 2016. Chen et al. used these data to conduct a study entitled “Association of body mass index and age with incident diabetes in Chinese adults: a population-based cohort study” (DOI: 10.1136/bmjopen-2018-021768.) and have deposited all study data in the Dryad Digital Repository.

### Definition of outcome

This retrospective cohort study had two primary follow-up outcomes: glycemic reversal in individuals with prediabetes or attainment of the study’s follow-up endpoint. Prediabetes was defined as a FPG concentration ranging from 5.6 to 6.9 mmol/L. Glycemic reversal was defined as an FPG level < 5.6 mmol/L during the follow-up period, with no self-reported diagnosis of DM (12).

### Study design and participants

**Figure 1:**
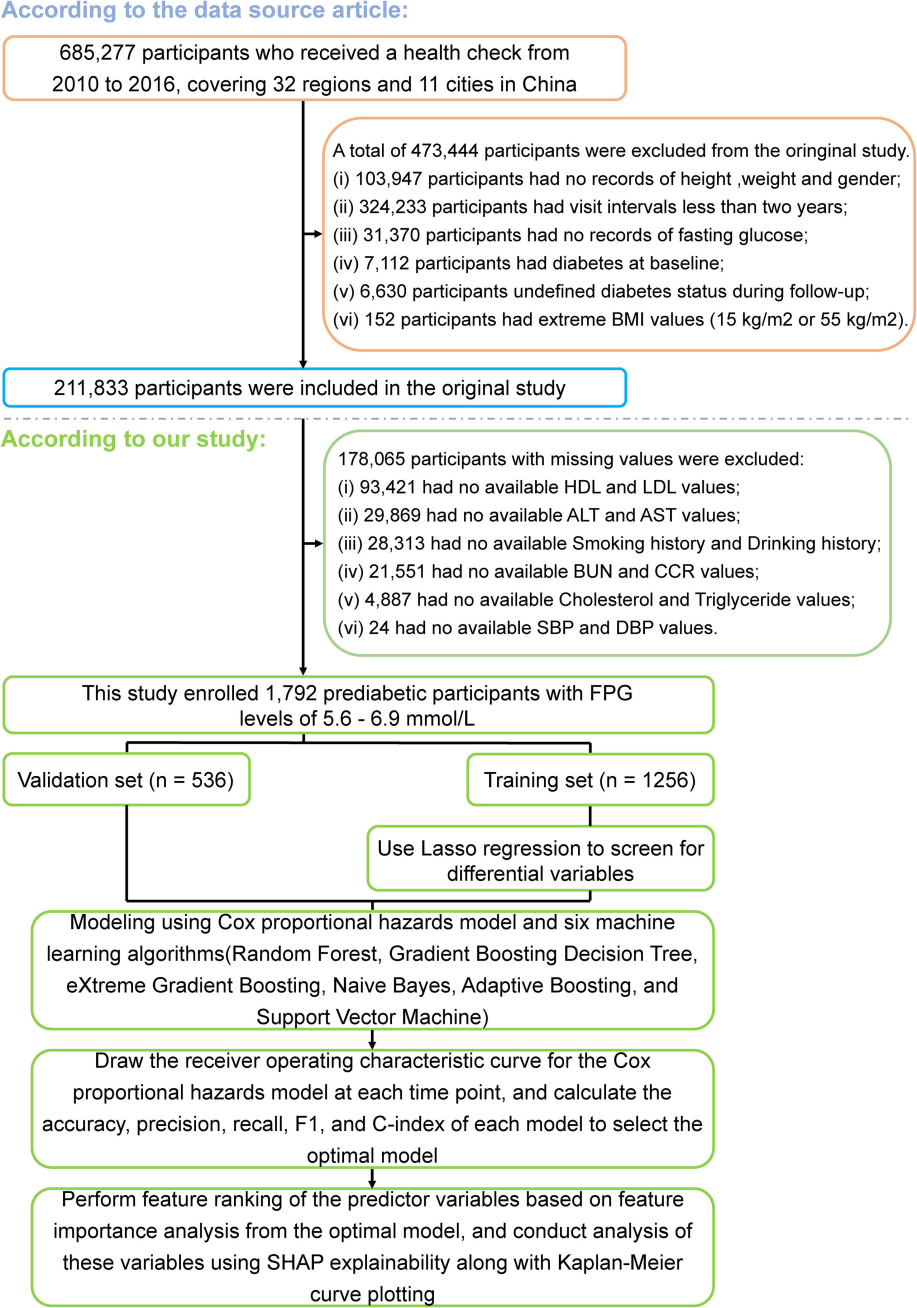
Flowchart of the study design and data analysis. BMI, body mass index; SBP, systolic blood pressure; DBP, diastolic blood pressure; HDL, high-density lipoprotein; LDL, low-density lipoprotein; ALT, alanine aminotransferase; AST, aspartate aminotransferase; BUN, blood urea nitrogen; CCR, creatinine clearance rate; FPG, fasting plasma glucose; SHAP, shapley additive explanations.

All participants in the raw data (n = 685,277) were aged ≥ 20 years and had undergone at least two health check-ups between 2010 and 2016. Of these, 7,112 were diagnosed with diabetes (2,997 via self-report and 4,115 via FPG ≥ 7.0 mmol/L). Participants were excluded if they had: unclear diabetes status during follow-up (n = 6,630); incorrectly recorded gender (n = 1); no height or weight measurements (n = 103,946); no FPG measurements (n = 31,370); BMI < 15 kg/m² or > 55 kg/m² (n = 152); or follow-up duration < 2 years (n = 324,233). A total of 211,833 participants were included in the original study. To develop a predictive model for glycemic reversal in individuals with prediabetes, participants with missing data from the original study were further excluded. This included those without recorded high-density lipoprotein (HDL) or low-density lipoprotein (LDL) levels (n = 93,421); alanine aminotransferase (ALT) or aspartate aminotransferase (AST) levels (n = 29,869); creatinine clearance rate (CCR) or blood urea nitrogen (BUN) levels (n = 21,551); triglyceride (TG) or total cholesterol (TC) levels (n = 4,887); systolic or diastolic blood pressure measurements (n = 24); or self-reported smoking or alcohol consumption history (n = 28,313). Ultimately, 1,792 participants who met the above criteria and had an FPG concentration between 5.6 and 6.9 mmol/L were included in the present study (**Figure 1**).

### Ethics statement

Study data were retrieved from Dryad, an open-access data repository. The original study obtained ethical approval from the Rich Healthcare Group Review Board and adhered to the tenets of the Declaration of Helsinki. In line with ethical guidelines, no additional ethical application was required for this secondary analysis. Furthermore, all data were anonymized, precluding the need for further informed consent (11). This study is a secondary analysis of the original research, involving only the extraction and analysis of raw data without any modifications to the original dataset.

### Feature selection

The enrollment time of 1,792 participants was defined as the date of their first medical visit. Each time participants attended the physical examination center, they completed a detailed questionnaire covering demographic information, lifestyle factors, and family history of chronic diseases. Trained physical examination physicians measured participants’ basic clinical parameters, including height, weight, and blood pressure. Biochemical indices were analyzed from venous blood samples collected after a minimum 10-hour fast, using an automatic analyzer (Beckman 5800). The tested parameters included FPG, TG, TC, HDL, LDL, CCR, BUN, ALT, and AST. BMI was calculated as weight divided by the square of height (kg/m²). All clinical data were collected following standardized procedures and under uniform conditions.

### Machine learning model development and evaluation

To investigate the feasibility of glycemic reversal in individuals with prediabetes and the differences among various influencing factors, we employed six machine learning algorithms: RF, gradient boosting decision tree (GBDT), XG Boost, naive Bayes (NB), adaptive boosting (Ada Boost), and SVM, along with the Cox proportional hazards model for model development. RF is an ensemble learning method that makes decisions by constructing multiple decision trees and aggregating their predictive outputs. Its core lies in the integration of randomness and ensemble strategies, which enhance the model’s predictive accuracy and generalization ability (13). GBDT is an ensemble learning algorithm based on the Boosting framework, operating by iteratively constructing multiple regression trees. Each new tree aims to predict the residuals left by the previous one. It uses the negative gradient of the loss function as the learning target for each new tree, gradually optimizes model performance via gradient descent, and ultimately obtains the output through weighted summation of predictive results from all trees (14). XG Boost is an efficient ensemble learning algorithm based on gradient-boosted decision trees, improving predictive accuracy by sequentially constructing decision trees that learn residuals. It is characterized by high performance, flexibility, interpretability, and wide applicability (15). NB is a probabilistic classification algorithm based on Bayes’ theorem, simplifying the calculation of joint probabilities through the naive assumption of feature independence and enabling efficient prediction of class labels. It is distinguished by fast training speed and suitability for large-scale data and multi-classification tasks (16). Ada Boost constructs a more powerful classifier by combining multiple weak classifiers through dynamic adjustment of sample weights. It features an adaptive weighting mechanism that enhances generalization ability, avoids overfitting, and is applicable to both binary and multi-classification tasks (17). SVM is a supervised learning algorithm whose core is finding the optimal hyperplane that maximizes the class margin for classification or regression. It can effectively handle non-linear problems via kernel tricks and ensures model generalization ability through the principle of structural risk minimization (18). The Cox proportional hazards model is a semi-parametric method for survival analysis, assessing the impact of independent variables on the hazard rate by comparing the order of event occurrence. Its core requirement is that the hazard ratio (HR) remains constant across different individuals. This model is particularly suitable for medical research with censored data and can analyze the independent effects of multiple prognostic factors on survival (19).

Six machine learning algorithms with predefined hyperparameters (**Supplementary Table S1**) were implemented in R. Data from 1792 participants were randomly split into a training set and a validation set at a 7:3 ratio using the createDataPartition function, with a random seed of 86413775 to ensure reproducibility. Both sets were further partitioned into five subsets via 5-fold cross-validation; each subset was used to train and test the aforementioned machine learning models, and the final performance metrics were reported as the mean of the five iterations. This approach enables robust assessment of the generalizability of each model. Receiver operating characteristic (ROC) curves were plotted for each model on both the training and validation sets to intuitively illustrate differences in their ability to discriminate between outcomes. Subsequently, model performance was evaluated on the validation set using six metrics: time-dependent area under the curve (t-AUC), accuracy, precision, recall, F1, and C-index. In survival analysis, t-AUC quantifies a model’s capacity to distinguish between distinct outcomes at specific time points, reflecting dynamic predictive accuracy. Accuracy represents the proportion of correctly predicted samples relative to the total, indicating overall predictive correctness. Precision denotes the proportion of true positive samples among all predicted positives, reflecting predictive precision. Recall is the proportion of true positives correctly identified, indicating the model’s ability to capture positive cases. The F1 is the harmonic mean of precision and recall, providing a balanced measure of overall performance. The C-index assesses the concordance between predicted risk scores and actual event times in survival analysis, reflecting the model’s ability to rank risk appropriately. These metrics comprehensively characterize the overall performance of each model and their utility in clinical decision-making, facilitating the identification of the algorithm with the optimal predictive efficacy (20).

Feature importance analysis was performed on the optimal model to determine the impact of feature variables on predicted outcomes. The importance of each feature variable was calculated based on the model’s internal mechanisms and visualized by plotting feature importance charts. Continuous variables with top-ranked importance scores from the optimal model were stratified, and Kaplan-Meier (KM) curves were generated for each variable to explore differences in the incidence of glycemic reversal across subgroups (21). Shapley additive explanations (SHAP) analysis was employed to interpret the results of the optimal machine learning model. This approach intuitively illustrates the direction of contribution of each feature variable to the model outputs and the cumulative contribution value of all feature variables. Furthermore, individual sample instance plots from SHAP analysis enable quantification of both the direction and magnitude of contribution of distinct feature variables to the outcome event in each individual sample (22).

### Statistical analysis

A total of 17 clinical predictors were included in this study. Sample size was calculated based on the principle of events per variable (EPV) ≥ 20, requiring a minimum of 340 outcome events to optimize model predictive performance and mitigate the risk of overfitting (23). Statistical analyses were performed using R software (version 4.3.0). For continuous variables, those following a normal distribution were presented as mean ± standard deviation (x̄ ± s) and compared between groups using the t-test. Variables with a skewed distribution were expressed as median and interquartile range [M (IQR)], with group comparisons conducted via the Mann-Whitney U test. Categorical variables were summarized as frequencies and percentages [n (%)], and group differences were assessed using the Chi-square test or Fisher’s exact test, as appropriate. In the training set, features with significant between-group differences were screened using least absolute shrinkage and selection operator (LASSO) regression. Six machine learning algorithms and a Cox regression model were then applied to the screened variables to construct predictive models for glycemic reversal in individuals with prediabetes. All analyses used two-tailed tests, with the significance level set at α = 0.05.

## Results

### Baseline characteristics of overall cohort data

1792 participants with prediabetes and complete baseline data, medical history, and laboratory test results were included in this study. The mean age of the participants was 46.80 ± 11.73 years, with 1318 males accounting for 73.5%. Among them, 28.0% had a smoking history, 27.2% had a drinking history, and 124 participants (6.9%) had a family history of diabetes. The mean follow-up duration for all participants was 2.73 ± 0.75 years, with a median follow-up of 2.68 (2.06, 3.05) years. After 5 years of follow-up, 52.6% (942/1792) of participants with prediabetes achieved glycemic reversal, indicating a high potential for glycemic reversal in this population (**Table 1**).

**Table 1:** Comparison of clinical indicators between participants with prediabetes and normoglycemia in the overall cohort data.

Variables	Total (n = 1792)	Prediabetes (n = 850)	Normoglycemia (n = 942)	P value
Male, n (%)	1318 (73.5%)	645 (75.9%)	673 (71.4%)	0.04
Smoking history, n (%)	502 (28.0%)	254 (29.9%)	248 (26.3%)	0.11
Drinking history, n (%)	487 (27.2%)	223 (26.2%)	264 (28%)	0.43
Family history of diabetes, n (%)	124 (6.9%)	67 (7.9%)	57 (6.1%)	0.15
Age, years	46.80 ± 11.73	50.23 ± 11.44	43.71 ± 11.11	<0.01
BMI, kg/m^2^	24.66 ± 3.22	25.16 ± 3.07	24.20 ± 3.29	<0.01
SBP, mmHg	123.90 ± 15.65	126.09 ± 16.34	121.93 ± 14.72	<0.01
DBP, mmHg	77.72 ± 10.38	79.14 ± 10.33	76.44 ± 10.27	<0.01
FPG, mmol/L	5.91 ± 0.30	6.00 ± 0.33	5.82 ± 0.24	<0.01
Cholesterol, mmol/L	5.00 ± 0.88	5.08 ± 0.87	4.93 ± 0.89	<0.01
Triglyceride, mmol/L	1.78 ± 1.32	1.90 ± 1.33	1.67 ± 1.31	<0.01
HDL, mmol/L	1.37 ± 0.29	1.36 ± 0.3	1.37 ± 0.28	0.34
LDL, mmol/L	2.87 ± 0.70	2.93 ± 0.70	2.83 ± 0.70	<0.01
ALT, U/L	28.69 ± 23.33	30.51 ± 27.3	27.04 ± 18.92	<0.01
AST, U/L	27.15 ± 11.51	27.89 ± 13.15	26.47 ± 9.76	0.01
BUN, mmol/L	5.01 ± 1.20	5.12 ± 1.21	4.91 ± 1.18	<0.01
CCR, μmol/L	74.87 ± 14.85	74.75 ± 14.6	74.97 ± 15.08	0.76

Data are shown as means ± standard deviation for normally distributed variables and percentages for categorical variables. BMI, body mass index; SBP, systolic blood pressure; DBP, diastolic blood pressure; FPG, fasting plasma glucose; HDL, high-density lipoprotein; LDL, low-density lipoprotein; ALT, alanine aminotransferase; AST, aspartate aminotransferase; BUN, blood urea nitrogen; CCR, creatinine clearance rate.

In the normoglycemic group, the proportion of males was 71.4% (673 participants), which was significantly higher than that in the prediabetic group. In contrast, levels of age, BMI, SBP, DBP, FPG, cholesterol, triglycerides, LDL, ALT, AST, and BUN were all significantly lower in the normoglycemic group compared with the prediabetic group, with statistically significant differences (P < 0.05) (**Table 1**). To develop a predictive model for glycemic reversal in individuals with prediabetes while ensuring the model’s generalizability, 1792 participants were randomly assigned to a training set (1256 participants, 70.1%) and a validation set (536 participants, 29.9%) at a 7:3 ratio. Intergroup difference analysis revealed no statistically significant differences in baseline characteristics, past medical history, or clinical laboratory findings between the training set and validation set (all P > 0.05), indicating that randomization effectively balanced confounding factors between the two groups (**Supplementary Table S2**). Further analysis of the training set demonstrated that age, BMI, SBP, DBP, FPG, cholesterol, triglycerides, LDL, ALT, AST, and BUN levels, as well as the prevalence of smoking history, were significantly lower in the normoglycemic group than in the prediabetic group (P < 0.05) (**Supplementary Table S3**). The characteristic differences in indicators between the two groups in the training set were consistent with those in the overall population, further validating the validity of the predictive variables.

### Variable selection and construction of Cox proportional hazards model

**Figure 2:**
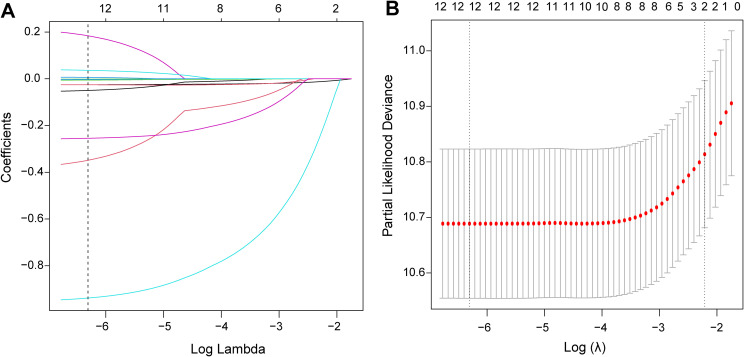
Use LASSO regression to screen for differential variables in the training set. **(A)** Variable coefficient path plot; **(B)** LASSO regression cross - validation curve. LASSO, least absolute shrinkage and selection operator.

To enhance the predictive accuracy of the forecasting model, we used LASSO regression in the training set to constrain the regression coefficients of variables, resulting in some coefficients tending to zero for feature selection and model simplification. In the LASSO regression coefficient path plot, each line represents one variable; the regression coefficient β of each variable in the model decreases with increasing penalty parameter λ, and the corresponding line compresses progressively toward the central zero line. As β continues to shrink and eventually reaches zero, the variable corresponding to that line contributes nothing to the model and is eliminated, thereby achieving variable selection. The x-axis denotes the penalty value, and the numbers above the plot indicate the number of variables retained in the model at the corresponding penalty value. LASSO regression generates a penalty function to shrink the regression coefficients of variables in the model, thereby avoiding overfitting and addressing multicollinearity (**Figure 2A**). Ten-fold cross-validation was used to determine the optimal regularization parameter λ, which identifies the key variables yielding the best model fit. The LASSO regression cross-validation plot illustrates changes in the model’s partial likelihood deviance across different λ values. The model achieves the best fit when the partial likelihood deviance is minimized, with the corresponding λ designated as lambda.min (**Figure 2B**). The variables selected at lambda.min and their respective regression coefficients are as follows: Age (-0.026), BMI (-0.026), SBP (-0.001), DBP (-0.003), FPG (-0.938), Cholesterol (0.184), Triglyceride (-0.050), LDL (-0.349), ALT (-0.006), AST (0.006), BUN (0.036), and Smoking history (-0.255) (**Supplementary Table S4**). The absolute value of the regression coefficient directly reflects the magnitude of the variable’s impact on glycemic reversal, and higher absolute values indicate a more pronounced effect.

**Figure 3:**
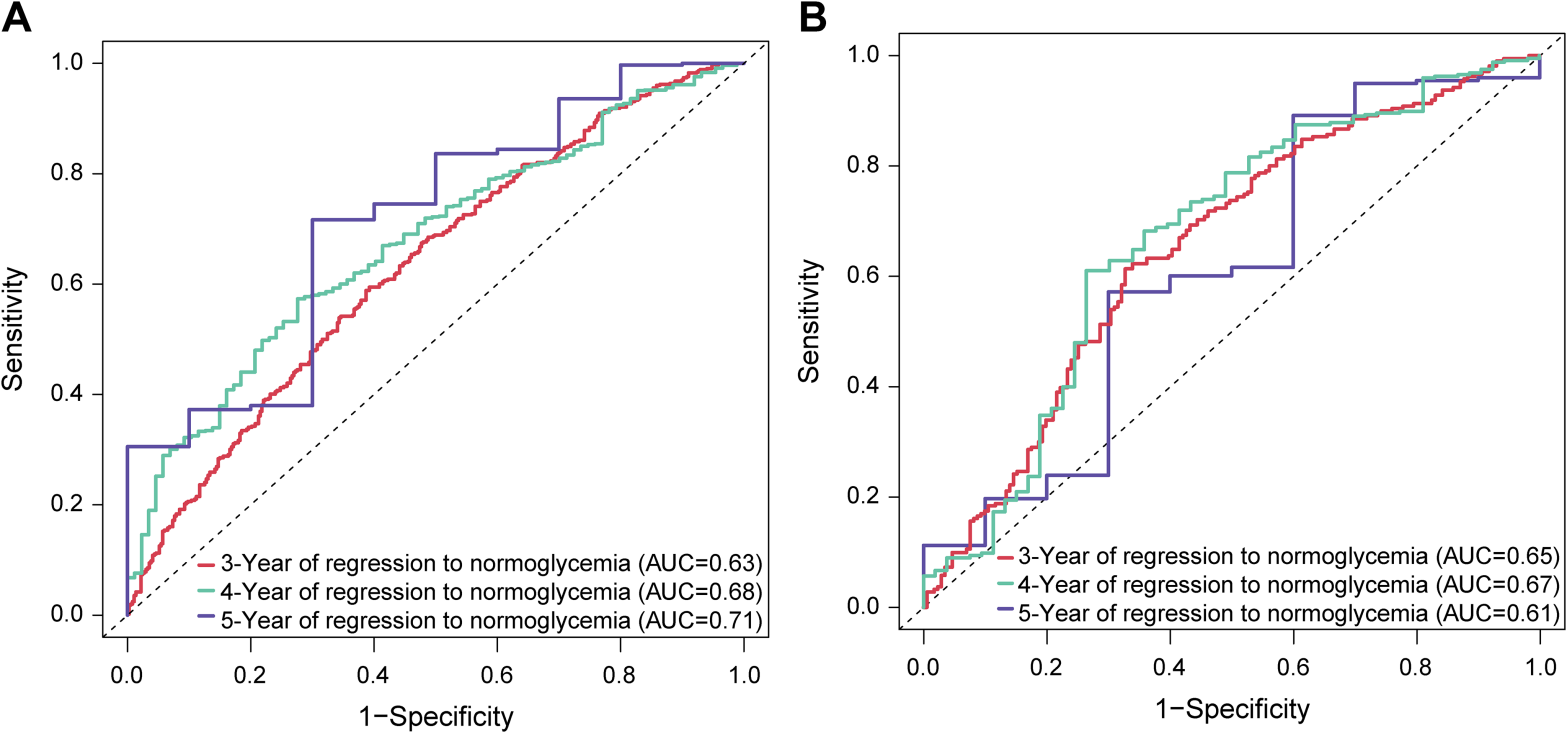
ROC curves for predicting glucose reversal at 3, 4, and 5 years in participants with prediabetes using a multivariate Cox proportional hazards model. **(A)** Based on the training set; **(B)** Based on the validation set. Cox, cox proportional hazards model; ROC, receiver operating characteristic.

Multivariate Cox regression analysis was performed with the occurrence of glycemic reversal at the end of follow-up as the dependent variable and the 12 feature variables selected by LASSO regression as independent variables. Results showed that Age (B: -0.026, HR: 0.97, 95% CI: 0.97 - 0.98), FPG (B: -0.961, HR: 0.38, 95% CI: 0.27 - 0.54), LDL (B: -0.399, HR: 0.67, 95% CI: 0.53 - 0.85), ALT (B: -0.007, HR: 0.99, 95% CI: 0.99 - 1.00), and Smoking history (B: -0.261, HR: 0.77, 95% CI: 0.64 - 0.93) were independent risk factors for blood glucose normalization in participants with prediabetes. In contrast, cholesterol (B: 0.228, HR: 1.26, 95% CI: 1.03 - 1.53) acted as a protective factor promoting glycemic reversal (**Table 2**). Time-dependent ROC curves were plotted for the training and validation sets at 3, 4, and 5 years of follow-up. In the training set, the AUCs for predicting glycemic reversal at 3, 4, and 5 years were 0.63, 0.68, and 0.71, respectively, indicating that the predictive performance of the model gradually improved with prolonged follow-up (**Figure 3A**). In the validation set, the AUCs for glycemic reversal at the same time points all exceeded 0.60, demonstrating good predictive consistency of the model (**Figure 3B**). Notably, in the multivariate Cox regression model, the AUCs of the ROC curves at 3, 4, and 5 years were all greater than 0.61 in both the training and validation sets. These findings confirm that the model has stable and effective predictive ability for glycemic reversal in the prediabetic population.

**Table 2:** Multivariate Cox proportional hazards model analysis of feature variables in the training set.

Variables	B	HR (95%CI)	P value
Age, years	-0.026	0.97 (0.97, 0.98)	<0.01
BMI, kg/m^2^	-0.025	0.98 (0.95, 1.00)	0.08
SBP, mmHg	-0.001	1.00 (0.99, 1.01)	0.73
DBP, mmHg	-0.003	1.00 (0.99, 1.01)	0.61
FPG, mmol/L	-0.961	0.38 (0.27, 0.54)	<0.01
Cholesterol, mmol/L	0.228	1.26 (1.03, 1.53)	0.03
Triglyceride, mmol/L	-0.059	0.94 (0.88, 1.01)	0.12
LDL, mmol/L	-0.399	0.67 (0.53, 0.85)	<0.01
ALT, U/L	-0.007	0.99 (0.99, 1.00)	0.05
AST, U/L	0.009	1.01 (0.99, 1.02)	0.25
BUN, mmol/L	0.042	1.04 (0.98, 1.11)	0.22
Smoking history, yes vs no	-0.261	0.77 (0.64, 0.93)	<0.01

BMI, body mass index; SBP, systolic blood pressure; DBP, diastolic blood pressure; FPG, fasting plasma glucose; LDL, low-density lipoprotein; ALT, alanine aminotransferase; AST, aspartate aminotransferase; BUN, blood urea nitrogen.

### Construction and performance comparison of six machine learning models

**Figure 4:**
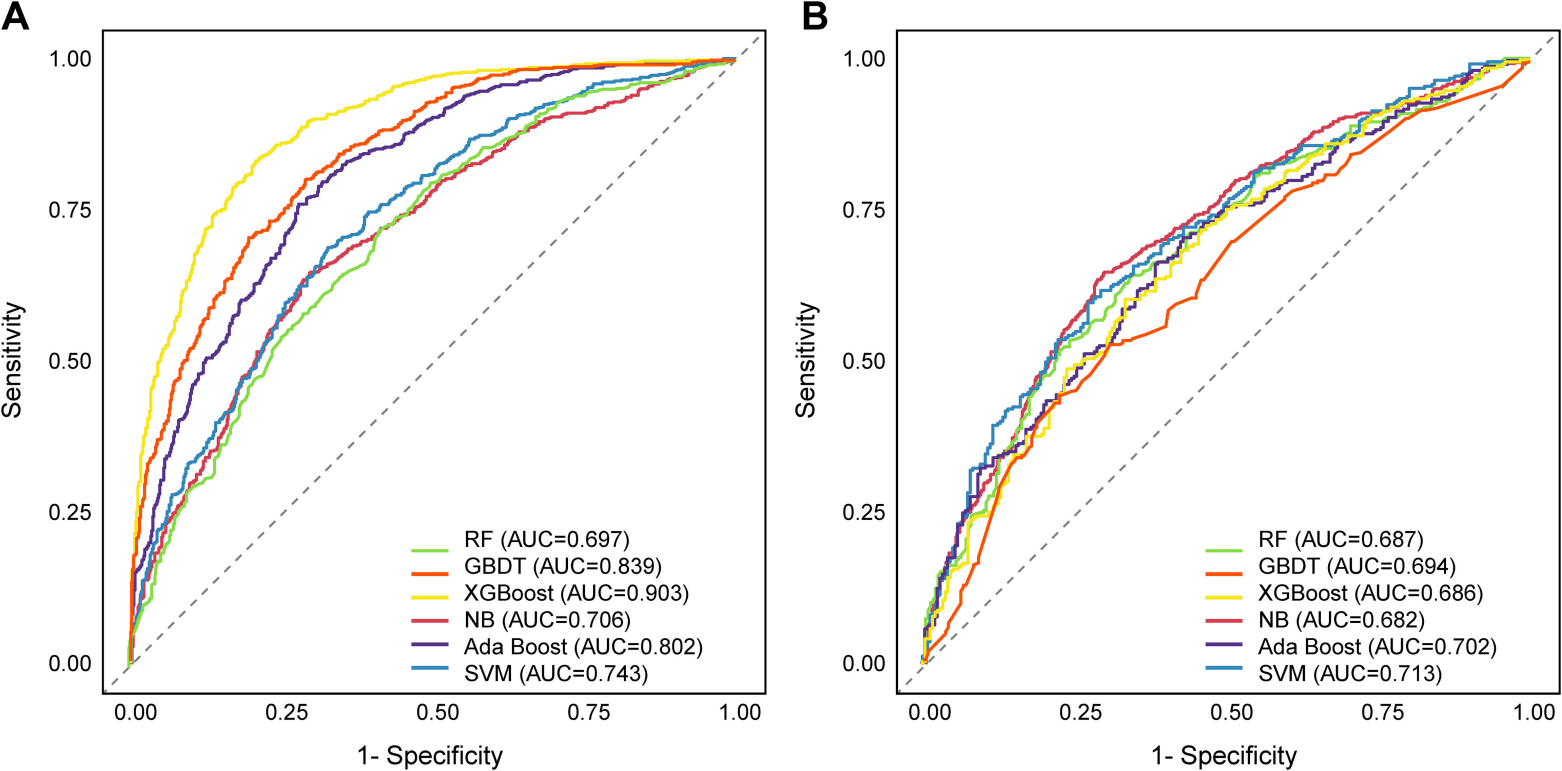
ROC curves for six machine learning algorithms predicting glucose reversal in participants with prediabetes. **(A)** Based on the training set; **(B)** Based on the validation set. ROC, receiver operating characteristic; RF, random forest; GBDT, gradient boosting decision tree; XG Boost, eXtreme gradient boosting; NB, naive bayes; Ada Boost, adaptive boosting; SVM, support vector machine.

To develop a machine learning model for predicting glycemic reversal in individuals with prediabetes, 12 feature variables selected by LASSO regression were incorporated into RF, GBDT, XG Boost, NB, Ada Boost, and SVM for model construction. In the training set, XG Boost achieved the highest AUC of 0.903, making it the top-performing model, while GBDT (AUC: 0.839) and Ada Boost (AUC: 0.802) followed closely with robust ROC performance. Subsequent models included SVM (AUC: 0.743), NB (AUC: 0.706), and RF (AUC: 0.697) (**Figure 4A**). However, when validating the models’ generalizability using the validation set, significant convergence and fluctuation in ROC performance were observed across all algorithms. XG Boost and GBDT, which led in the training set, showed a substantial decline in AUC in the validation set (XGBoost: 0.686; GBDT: 0.694). RF and NB also experienced a modest reduction in AUC (RF: 0.687; NB: 0.682). In contrast, SVM demonstrated greater stability in ROC performance in the validation set, achieving the highest AUC of 0.713, and Ada Boost ranked second (AUC: 0.702); all other models yielded an AUC below 0.70 (**Figure 4B**).

Further quantitative evaluation of the multidimensional performance of seven models was conducted on the validation set. The SVM achieved a t-AUC of 0.711, ranking first among all models. Its accuracy (0.652), precision (0.620), recall (0.661), and F1 (0.639) also reached above-average levels. Meanwhile, the C-index of SVM was 0.709 (95% CI: 0.632 - 0.784), which was similarly superior to the other six predictive models (**Table 3**). Considering the performance changes from the training set to the validation set, as well as the multi-metric performance on the validation set, SVM exhibited superior generalization ability and overall predictive performance in forecasting glycemic reversal outcomes among individuals with prediabetes.

**Table 3:** Performance evaluation of six machine learning algorithms and the Cox proportional hazards model for constructing a model to predict glucose reversal in participants with prediabetes.

Model	t-AUC	Accuracy	Precision	Recall	F1	C-Index
RF	0.687	0.651	0.609	0.617	0.613	0.687 (0.642, 0.731)
GBDT	0.688	0.636	0.590	0.613	0.601	0.687 (0.642, 0.732)
XG Boost	0.689	0.651	0.604	0.642	0.622	0.688 (0.643, 0.731)
NB	0.682	0.634	0.609	0.513	0.557	0.681 (0.634, 0.726)
Ada Boost	0.702	0.644	0.601	0.608	0.605	0.701 (0.656, 0.745)
SVM	0.711	0.652	0.620	0.661	0.639	0.709 (0.632, 0.784)
Cox	0.651	0.662	0.681	0.647	0.663	0.648 (0.626, 0.676)

RF, random forest; GBDT, gradient boosting decision tree; XG Boost, eXtreme gradient boosting; NB, naive bayes; Ada Boost, adaptive boosting; SVM, support vector machine. t-AUC, time-dependent area under the curve. Cox, cox proportional hazards model; CI, confidence interval.

### Ranking and explanation of the importance of feature variables for the optimal model

**Figure 5:**
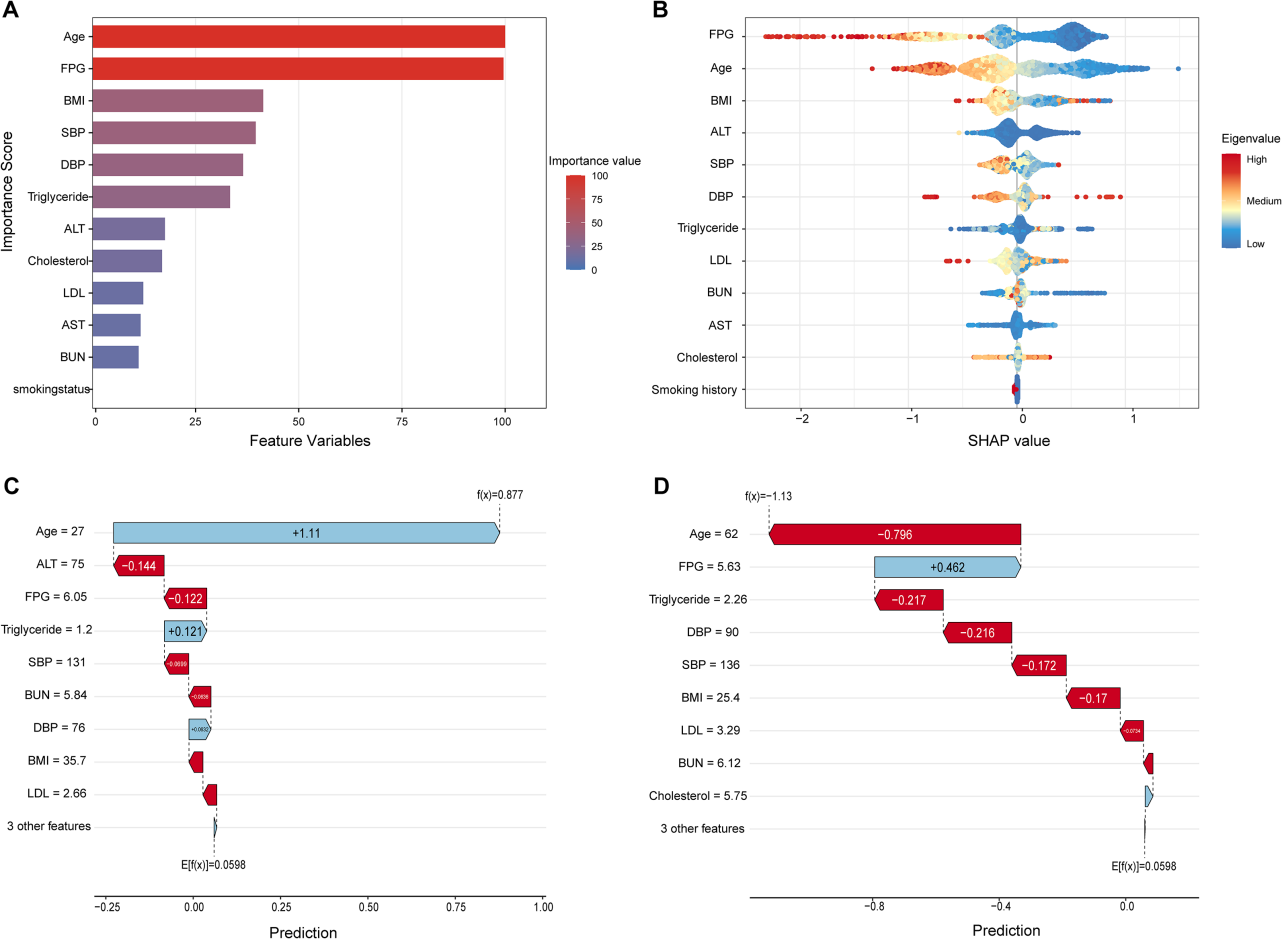
Feature variable importance ranking and interpretation of feature variables using SHAP analysis. **(A)** Feature variable importance ranking chart; **(B)** SHAP scatter plot; **(C)** SHAP force plot for samples prone to glycemic reversal; **(D)** SHAP force plot for samples not prone to glycemic reversal. SHAP, shapley additive explanations; BMI, body mass index; SBP, systolic blood pressure; DBP, diastolic blood pressure; FPG, fasting plasma glucose; LDL, low-density lipoprotein; ALT, alanine aminotransferase; AST, aspartate aminotransferase; BUN, blood urea nitrogen.

Among the comparisons of six machine learning models and the Cox proportional hazards model for predicting glycemic reversal, the SVM demonstrated excellent discriminative ability and balance. Accordingly, we ranked the 12 feature variables using the built-in feature importance analysis of SVM. Age, FPG, BMI, SBP, DBP, and Triglyceride exhibited high contribution to the SVM-based prediction of glycemic reversal (**Figure 5A**). Subsequently, SHAP explainability analysis was employed to interpret the model’s prediction logic and quantify the contribution intensity and direction of each feature to the model output. The explainability analysis included SHAP summary plots and SHAP force plots. In the SHAP summary plot, elevated FPG, increased Age, and higher BMI (predominantly red, representing high feature values) exerted a negative contribution to the SVM-predicted probability of glycemic reversal, with higher values of these features reducing the predicted likelihood of reversal. In contrast, lower values of ALT, SBP, and Triglyceride (predominantly blue, representing low feature values) showed a positive contribution, as lower values of these features increased the predicted probability of glycemic reversal (**Figure 5B**). The force plots illustrated the prediction logic for specific samples in the prediabetic population that were predicted by the model to be either prone or not prone to glycemic reversal. Here, f(x) denotes the predicted probability of glycemic reversal for a given sample. A positive f(x) indicates a high predicted probability of glycemic reversal, while a negative f(x) indicates a low predicted probability. The length of the arrow corresponding to each feature reflects the intensity of its impact on the model’s prediction, with longer arrows indicating stronger contributions. Red arrows represent a negative contribution to the predicted glycemic reversal, and blue arrows represent a positive contribution. In **Figure 5C**, f(x) was 0.877, indicating a high predicted likelihood of glycemic reversal for this sample; Age of 27 years and triglyceride level of 1.2 mmol/L were the primary feature contributors to this prediction. Conversely, the sample in **Figure 5D** had an f(x) of -1.13, with the model predicting a low probability of glycemic reversal. Age of 62 years, triglyceride level of 2.26 mmol/L, and DBP of 90 mmHg were the core feature contributors to this outcome.

**Figure 6:**
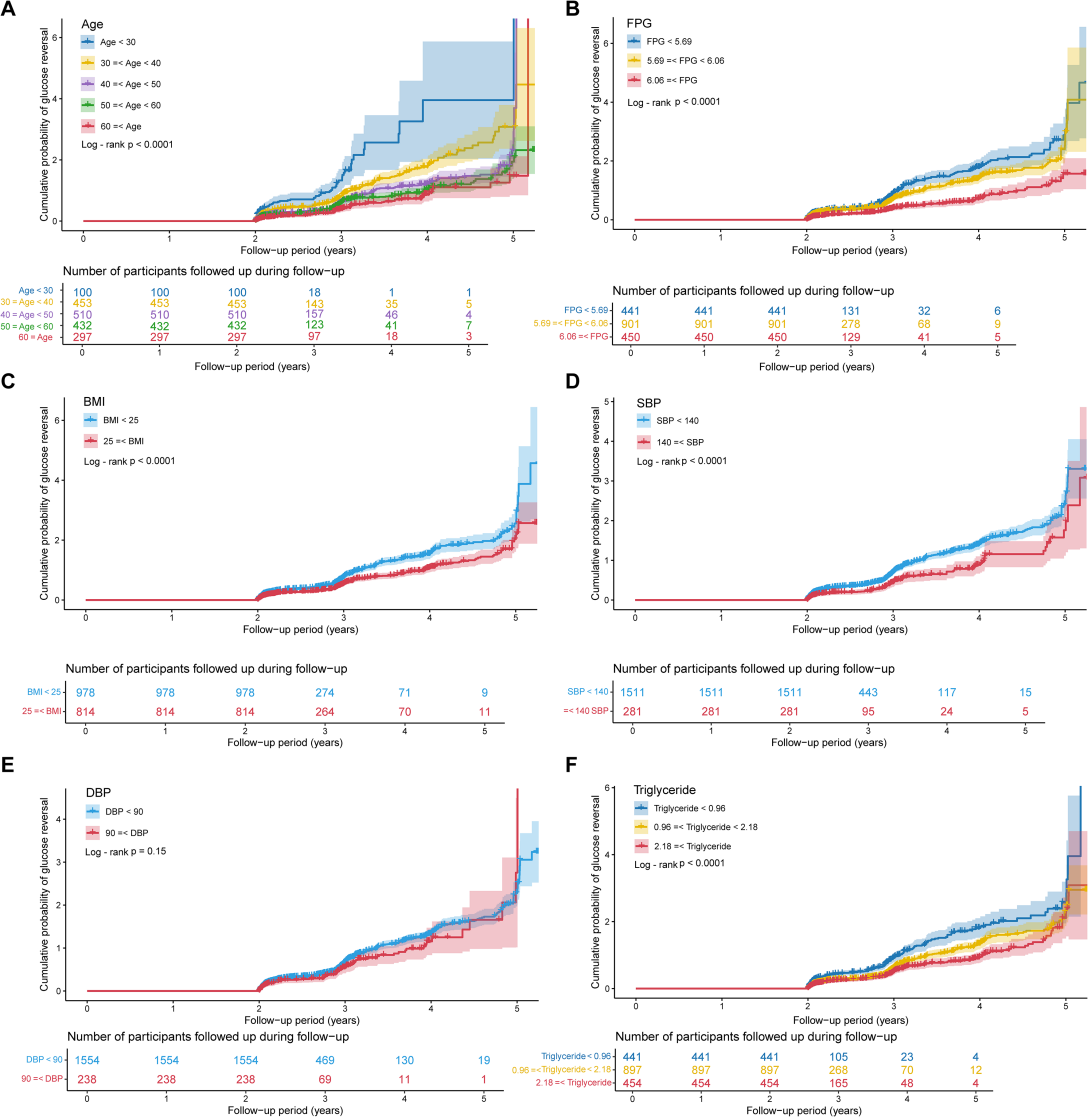
Kaplan - Meier curves for the top six most important feature variables. **(A)** Kaplan-Meier curve for age; **(B)** Kaplan-Meier curve for FPG; **(C)** Kaplan-Meier curve for BMI; **(D)** Kaplan-Meier curve for SBP; **(E)** Kaplan-Meier curve for DBP; **(F)** Kaplan-Meier curve for triglyceride. BMI, body mass index; SBP, systolic blood pressure; DBP, diastolic blood pressure; FPG, fasting plasma glucose.

To translate the core predictive features identified by SVM into a stratified assessment tool for glycemic reversal in individuals with prediabetes, this study performed stratified subgroup analyses of age, FPG, BMI, SBP, DBP, and triglycerides. KM curves were plotted to illustrate the cumulative probability of glycemic reversal across different subgroups, with intergroup differences compared using the log-rank test (**Figure 6**). In age stratification, the subgroup aged < 30 years exhibited a significantly higher cumulative probability of glycemic reversal than other subgroups, and this probability showed a gradual downward trend with increasing age (P < 0.001). For FPG stratification, the subgroup with FPG < 5.69 mmol/L maintained a consistently high cumulative reversal probability, whereas the subgroup with FPG ≥ 6.06 mmol/L had the lowest probability (P < 0.001). BMI was categorized into two subgroups: < 25 kg/m² and ≥ 25 kg/m². During follow-up, the cumulative reversal probability in the BMI < 25 kg/m² subgroup remained persistently higher than that in the BMI ≥ 25 kg/m² subgroup (P < 0.001). The subgroup with SBP < 140 mmHg had a significantly higher cumulative reversal probability than subgroups with higher SBP, and the probability decreased with increasing SBP levels (P < 0.001). KM curves for DBP stratification showed no obvious separation in cumulative reversal probability among subgroups, with no statistically significant intergroup difference (P = 0.15). Triglycerides were divided into three subgroups: < 0.96 mmol/L, 0.96 - 2.18 mmol/L, and ≥ 2.18 mmol/L. The subgroup with triglycerides < 0.96 mmol/L had a significantly higher cumulative reversal probability than the other two subgroups (P < 0.001). Collectively, these results indicate that five easily accessible indicators, namely age < 30 years, FPG < 5.69 mmol/L, BMI < 25 kg/m², SBP < 140 mmHg, and triglycerides < 0.96 mmol/L, can be used to rapidly identify individuals with prediabetes who have a high probability of glycemic reversal. In contrast, individuals with these indicators in the high-value ranges have a relatively low cumulative probability of glycemic reversal and should be prioritized for health education and lifestyle interventions.

## Discussion

Prediabetes refers to a state of impaired glucose regulation. Despite variations in diagnostic cutoffs across countries, its global prevalence ranges from 8% to 58% (24). Prediabetes is strongly associated with a significantly increased risk of cardiovascular disease, chronic kidney disease, cancer, and all-cause mortality, while restoration of normal blood glucose levels can substantially mitigate these risks (25). Identifying key factors that influence blood glucose changes in individuals with prediabetes is therefore crucial. Using data from a Chinese retrospective cohort study, we developed a predictive model for glycemic reversal in participants with prediabetes via the Cox proportional hazards model. Our findings indicated that age, FPG, cholesterol, LDL, ALT, and smoking history are major risk factors inhibiting glycemic reversal. Additionally, we analyzed feature variables using six machine learning algorithms. Comprehensive evaluation revealed that the SVM outperformed other machine learning models as well as the Cox proportional hazards model in terms of generalization performance. Variable importance analysis based on the SVM model demonstrated that age, FPG, BMI, SBP, DBP, and triglycerides are key factors influencing blood glucose changes in individuals with prediabetes. Identification of these critical factors facilitates the implementation of targeted interventions for this population, thereby promoting the restoration of normal blood glucose levels and reducing the risk of adverse outcomes such as progression to overt diabetes.

Prediabetes represents a critical reversible stage of glucose metabolism disorders, where glycemic reversal can be achieved through lifestyle interventions such as diet modification and physical activity. Once this stage progresses to T2DM, β-cell function damage is mostly irreversible, requiring long-term pharmacological control with a continuous elevation in the risk of complications (26). In studies investigating key factors influencing the recovery of normal blood glucose in individuals with prediabetes, the integration of the Cox proportional hazards model with cohort data overcomes the inherent limitation of traditional cross-sectional studies in depicting dynamic processes. This model quantifies the time-dependent effects of various risk factors on glycemic reversal events, clarifying the strength and direction of associations between characteristic variables and glucose recovery (27). Yi et al. (28), using retrospective cohort data from 15,415 participants, demonstrated that 43.0% of individuals with prediabetes achieved normal blood glucose after a median follow-up of 3.2 years. After adjusting for covariates including age, sex, and BMI via the Cox proportional hazards model, precise quantification revealed that metabolic syndrome-related insulin resistance (MetS-IR) had the strongest association with glycemic reversal (HR: 0.79) and optimal predictive performance. This provides evidence that systemic IR is a core regulatory target for glycemic reversal. However, the application of the Cox proportional hazards model in this study is constrained by its inherent characteristics: it assumes linear associations with covariates, requires adherence to the proportional hazards assumption, cannot automatically capture complex interaction effects of multiple factors, and relies on manual control of multicollinearity when handling high-dimensional data. To further enhance the automated efficiency of feature selection and the accuracy of complex pattern recognition, multiple machine learning algorithms can be employed to develop complementary strategies (29). Ensemble learning models, including RF, GBDT, XG Boost, and Ada Boost, can automatically explore nonlinear associations and complex interactions between covariates through an ensemble mechanism of multiple base learners, without being restricted by the linear and proportional hazards assumptions of the Cox model (30). SVM leverages kernel function mapping to efficiently fit nonlinear relationships in high-dimensional feature spaces and is naturally robust to multicollinearity (31). NB, centered on a probabilistic generative model, exhibits low computational complexity in high-dimensional data scenarios and eliminates the need for manual intervention in addressing multicollinearity (32). In the present study, multi-timepoint ROC curves of the Cox proportional hazards model were generated. Results showed that the AUC at 3, 4, and 5 years exceeded 0.61 in both the training and validation sets, indicating stable and effective predictive performance for glycemic reversal in individuals with prediabetes. Subsequently, 12 characteristic variables selected via LASSO regression were incorporated into RF, GBDT, XG Boost, NB, Ada Boost, and SVM to construct predictive models. In the training set, the AUC values of XG Boost (0.903), GBDT (0.839), Ada Boost (0.802), and SVM (0.743) were all higher than those of the Cox proportional hazards model. After validating generalization ability using the validation set, the ROC performance of each machine learning model remained significantly superior to that of the Cox model. In conclusion, the Cox proportional hazards model exhibits temporal stability and population applicability in predicting glycemic reversal, serving as a benchmark for such predictive tasks. Nevertheless, the six machine learning models demonstrate superior predictive accuracy and generalization ability, along with greater potential for external translation.

Notably, the top-performing XG Boost and GBDT in the training set exhibited substantial drops in AUC in the validation set (XG Boost: 0.686; GBDT: 0.694). The AUCs of RF and NB also decreased slightly (RF: 0.687; NB: 0.682), whereas the ROC curve of SVM demonstrated greater stability in the validation set, achieving the highest AUC of 0.713. As ensemble models based on the Boosting framework, XG Boost and GBDT iteratively focus on hard-to-classify samples in the training set and minimize training error. This inherent characteristic renders them prone to overlearning random noise specific to the training set and spurious associations between features and outcomes, thereby increasing overfitting risk and ultimately impairing generalizable predictive performance (33, 34). After conducting multidimensional quantitative evaluations of the seven models in the validation set, we found that SVM achieved the highest t-AUC of 0.711 among all models. Its accuracy (0.652), precision (0.620), recall (0.661), and F1 (0.639) all ranked in the upper-middle range. Furthermore, SVM’s C-index was 0.709 (95% CI: 0.632 - 0.784), which also outperformed the other six predictive models. Based on the principle of structural risk minimization, SVM balances fitting accuracy and generalization ability by maximizing the classification margin. Its built-in penalty parameter directly regulates overfitting risk, effectively preventing excessive adaptation to noise in the training set (35). Thus, SVM can capture the nonlinear associations between features and glycemic reversal without overly complex hyperparameter tuning. In the low-to-moderate dimensional setting of this study (12 features), it avoids the dilemma of over-segmentation arising from limited feature dimensions. To further enhance the model’s clinical utility and translational feasibility and to facilitate its practical application and dissemination in clinical settings, we ranked the 12 feature variables using SVM’s built-in feature importance analysis. The results indicated that age, FPG, BMI, SBP, DBP, and triglyceride contributed the most to SVM’s prediction of glycemic reversal outcomes.

Age ranks first in the analysis of factors influencing glycemic reversal in prediabetes. In individuals under 30 years of age, metabolic regulatory mechanisms have not sustained irreversible organic damage, with pancreatic β-cells showing no significant decline or apoptosis but only transient functional impairment, thereby retaining strong repair potential. Meanwhile, insulin resistance in target organs such as skeletal muscle and the liver is mostly induced by short-term unhealthy lifestyles without forming pathological structural remodeling, and insulin sensitivity can be rapidly restored through targeted interventions (36, 37). Consistent with this, our study found that the cumulative probability of glycemic reversal was significantly higher in the subgroup aged < 30 years than in other age groups. For this age cohort, measures including optimized dietary structure, regular aerobic exercise, and adjusted sleep rhythms can effectively reduce body weight, improve lipid metabolism, eliminate the core driving factors of prediabetes, and ultimately achieve physiological recovery of glucose regulatory mechanisms to reverse elevated blood glucose (38). A study by Alizadeh et al. (39) demonstrated a significant negative correlation between age and glycemic reversal in prediabetes, with individuals under 45 years having a notably higher reversal probability than those over 65 years. With advancing age, pancreatic β-cell function gradually declines; insulin synthesis and secretion capacity in middle-aged and elderly populations may decrease by approximately 30%-50% compared with adolescence (40). Additionally, β-cell sensitivity to glucose-dependent insulinotropic polypeptide (GLP-1) decreases, impairing GLP-1’s role in stimulating insulin secretion (41). Age-related loss of muscle mass in older adults also leads to reduced insulin receptor density and impaired glucose transporter type 4 (GLUT4) transport activity. Furthermore, free fatty acids released by visceral fat infiltrate the liver, affecting hepatic function and activating the protein kinase C epsilon (PKC-ϵ) pathway, which blocks insulin signal transduction and thereby elevates blood glucose. In our study, ALT and AST were also identified as important factors influencing glycemic reversal, supporting this mechanism. For elderly individuals with prediabetes, increasing protein and dietary fiber intake while reducing refined carbohydrates and saturated fats can balance nutrition and reduce visceral fat accumulation. Maintaining mild to moderate intensity exercise daily helps preserve muscle mass in older adults, creating conditions for glycemic reversal (40, 42). Studies have also suggested that α-glucosidase inhibitors (AGIs) may be beneficial for glycemic reversal in prediabetic individuals over 50 years of age (24). Therefore, full attention to the impact of age differences on glycemic reversal in prediabetes and development of individualized intervention strategies for different age groups are expected to more efficiently promote the reversal of blood glucose to normal levels.

As core metabolic abnormalities, hyperglycemia, hypertension, dyslipidemia, and obesity not only significantly increase the risk of progression to diabetes in individuals with prediabetes but also play a crucial role in glycemic reversal (43). Our study identified that FPG, SBP, DBP, BMI, and triglycerides are highly influential factors in predicting glycemic reversal in this population. As a core diagnostic criterion for diabetes, FPG directly reflects the pathological mechanisms of IR and β-cell dysfunction. Even when FPG levels in prediabetic individuals are below the diagnostic threshold for diabetes, values above the normal range can accelerate β-cell functional decline through glucotoxicity (44). A 4-year follow-up study demonstrated that prediabetic individuals with a baseline FPG closer to the lower limit of the prediabetes diagnostic criteria (5.6 mmol/L) are more likely to achieve glycemic reversal. In contrast, the probability of reversal decreases significantly as FPG approaches the diabetes diagnostic threshold (7.0 mmol/L) (45). Our stratified analysis further confirmed this association: the cumulative probability of glycemic reversal remained consistently high in the subgroup with FPG < 5.69 mmol/L, while the lowest cumulative reversal probability was observed in the subgroup with FPG ≥ 6.06 mmol/L. In a prospective cohort study, Liu et al. (46) showed that prediabetic individuals diagnosed solely by FPG who achieve glycemic reversal within 2 years have a significantly lower risk of subsequent cardiovascular and cerebrovascular events and all-cause mortality compared to those who progress to diabetes.

Hypertension directly damages the vascular endothelium via mechanical stress, activating the renin-angiotensin-aldosterone system (RAAS) and the oxidative stress-inflammatory cascade. This induces abnormal serine phosphorylation of insulin receptor substrate-1 (IRS-1) and inhibition of the phosphatidylinositol 3-kinase (PI3K)/Akt signaling pathway, leading to a significant reduction in insulin sensitivity (47). Studies have demonstrated that the Homeostasis Model Assessment of Insulin Resistance (HOMA-IR) is significantly elevated in individuals with hypertension, showing a positive correlation with FPG and postprandial blood glucose (r = 0.32). Furthermore, when hypertension coexists with high HOMA-IR, the risk of progression from prediabetes to diabetes increases by 6.78-fold (48). In the differential analysis of the present study, both SBP and DBP were within the normal range in both the prediabetes and normoglycemia groups, but the mean values were higher in the prediabetes group. A study involving 40,046 participants showed that for every approximately 10 mmHg reduction in SBP among individuals with prediabetes, the probability of blood glucose normalization increases by 10% (49). The optimal SBP range for individuals with prediabetes is 120–130 mmHg. However, when SBP is below 100 mmHg or above 150 mmHg, it disrupts glucose homeostasis by exacerbating insulin resistance, vascular endothelial damage, and inflammatory responses. This not only reduces the likelihood of glycemic reversal but also increases the risk of all-cause mortality (42). Consistently, we found that the probability of glycemic reversal was significantly higher in prediabetic individuals with SBP < 140 mmHg compared with those with SBP ≥ 140 mmHg, and this probability decreased with increasing SBP levels.

In the SHAP summary plot, an increase in BMI exerted a negative contribution to the probability of glycemic reversal predicted by the SVM. BMI is a key reference indicator for assessing body fatness, with its levels positively correlated with the degree of obesity (50). In our study, the mean BMI of the prediabetes group was 25.16 kg/m², which falls within the overweight category. In prediabetic individuals with excessively high BMI, excess accumulated adipose tissue releases free fatty acids, lipotoxic metabolites, and inflammatory cytokines such as tumor necrosis factor-alpha (TNF-α) and Interleukin-6 (IL-6). These substances reduce insulin sensitivity in target organs including the liver and skeletal muscle, interfere with signaling pathways subsequent to insulin-receptor binding, and thereby compel pancreatic β-cells to initiate compensatory hyperinsulinemia to maintain systemic glucose homeostasis (51). Prolonged secretory overload induces dedifferentiation, apoptosis, and impaired secretory function of pancreatic β-cells. When the secretory capacity of pancreatic β-cells fails to compensate for IR, the glucose regulatory mechanism becomes completely disrupted, facilitating the progression of prediabetes to T2DM (52). Furthermore, a BMI ≥ 24 kg/m² is a key risk factor for elevated triglycerides and abnormal cholesterol metabolism. Excess fat accumulation promotes the massive synthesis and accumulation of triglycerides by enhancing hepatic fatty acid synthesis and inhibiting lipolysis; it also interferes with reverse cholesterol transport, thereby leading to elevated cholesterol levels (53, 54). The lipotoxic effects of triglycerides not only exacerbate IR further but also directly impair pancreatic β-cell function (55). Therefore, implementing intervention measures such as a balanced diet and regular physical activity in prediabetic individuals to optimize BMI, reduce triglyceride levels, regulate total cholesterol, alleviate IR, and protect pancreatic β-cell function is crucial for restoring normal glucose homeostasis (56).

However, this study has several limitations. First, the study population was restricted exclusively to individuals of Chinese ethnicity. To develop a predictive model for glycemic reversal in people with prediabetes, participants with missing values in the original dataset were excluded, a procedure that may have introduced selection bias. Therefore, future studies could reduce the rate of data missingness by conducting multicenter collaborations and optimizing data collection and quality control protocols. Simultaneously, enrolling study participants from diverse countries and ethnicities would enhance the external applicability and generalizability of the model, while further exploring the core factors influencing glycemic reversal in prediabetic populations across different demographic contexts. Second, the diagnosis of prediabetes in this study relied solely on the criterion of FPG levels ranging from 5.6 to 6.9 mmol/L, with no incorporation of 2-h PG or A1C results. This approach potentially poses a risk of underestimation in the screening of prediabetic individuals. Nevertheless, implementing comprehensive 2-h PG or A1C testing for large-scale study populations presents substantial practical challenges in real-world settings. Finally, this study was a secondary analysis of a public database, which, constrained by the inherent attributes of the original data, could not be dynamically updated. Consequently, information regarding participants’ medication regimens, educational attainment, and lifestyle factors including diet and physical activity was unavailable. It is worth noting, however, that the key factors identified in this study as influencing blood glucose outcomes in prediabetic individuals are consistent with the findings of previous research. This consistency, in turn, corroborates the reference value and applicability of our results in research related to glycemic reversal.

## Conclusions

Based on retrospective cohort data from China, we identified through machine learning algorithms and Cox regression models that age, FPG, BMI, SBP, DBP, and triglycerides are key factors influencing glycemic reversal in individuals with prediabetes. Among these, age was the most prominent determinant, with younger individuals showing a significantly higher probability of glycemic reversal than older adults. Nevertheless, older adults can still achieve effective glycemic improvement through a balanced diet, scientific nutrient supplementation, and moderate physical activity. For metabolic indicators including blood lipids, blood pressure, and BMI, reduction to normal ranges via lifestyle interventions and personalized treatment regimens contributes substantially to glycemic recovery. Clarifying the impact of these factors not only provides precise targets for the early intervention of prediabetes but also offers reliable predictive evidence and preventive insights for clinicians, thereby advancing the overall level of prediabetes prevention and control.

## Data Availability

Publicly available datasets were analyzed in this study. This data can be found here: The raw data involved in this study are available in the Dryad database (https://www.datadryad.org/stash).
