# Supplementary material for: A predictive study of glycaemic reversal in Chinese individuals with prediabetes based on machine learning: a 5-year cohort study
Source: Front Endocrinol (Lausanne). 2026 Jan 28;17:1686082. doi: 10.3389/fendo.2026.1686082 (PMC12890694; doi:10.3389/fendo.2026.1686082)
Supplement: Supplementary file 1 [file Table1.docx]

**S Table 1.** Hyperparameters of random forest, gradient boosting decision tree, eXtreme gradient boosting, naive bayes, adaptive boosting and support vector machine.

| Model | Hyperparameter name | Hyperparameter value |
| --- | --- | --- |
| RF | mtry | floor(sqrt(length(predictors))) |
|  | ntree | 500 |
|  | importance | TRUE |
|  |  |  |
| GBDT | n.trees | 100 |
|  | interaction.depth | 3 |
|  | shrinkage | 0.1 |
|  | n.minobsinnode | 10 |
|  |  |  |
| XGBoost | nrounds | 100 |
|  | max_depth | 3 |
|  | eta | 0.1 |
|  | gamma | 0 |
|  | colsample_bytree | 0.8 |
|  | min_child_weight | 1 |
|  | subsample | 0.8 |
|  | objective | binary:logistic |
|  |  |  |
| NB | laplace | 1 |
|  | usekernel | FALSE |
|  | adjust | 1 |
|  |  |  |
| AdaBoost | mfinal | 100 |
|  | coeflearn | Zhu |
|  | maxdepth | 2 |
|  |  |  |
| SVM | C | 0.1,1,10 (grid search) |
|  | sigma | 0.001,0.01,0.1 (grid search) |
|  | kernel | svmRadial |

RF, random forest; GBDT, gradient boosting decision tree; XG Boost, eXtreme gradient boosting; NB, naive bayes; Ada Boost, adaptive boosting; SVM, support vector machine. t - AUC, time - dependent area under the curve. Cox, cox proportional hazards model; CI, confidence interval.
