# Supplementary material for: A predictive study of glycaemic reversal in Chinese individuals with prediabetes based on machine learning: a 5-year cohort study
Source: Front Endocrinol (Lausanne). 2026 Jan 28;17:1686082. doi: 10.3389/fendo.2026.1686082 (PMC12890694; doi:10.3389/fendo.2026.1686082)
Supplement: Supplementary file 2 [file Table2.docx]

**S Table 2. Comparison of clinical indicators between the training set and the validation set.**

| Variables | validation set  (n=536) | Training set  (n=1256) | P value |
| --- | --- | --- | --- |
| Male, n (%) | 393 (73.3 %) | 925 (73.6 %) | 0.93 |
| Smoking history, n (%) | 137 (25.6 %) | 365 (29.1 %) | 0.15 |
| Drinking history, n (%) | 141 (26.3 %) | 346 (27.5 %) | 0.63 |
| Family history of diabetes, n (%) | 42 (7.84 %) | 82 (6.53 %) | 0.37 |
| Age, years | 46.8 ± 11.60 | 46.8 ± 11.80 | 0.93 |
| BMI, kg/m2 | 24.6 ± 3.32 | 24.7 ± 3.18 | 0.40 |
| SBP, mmHg | 124 ± 15.70 | 124 ± 15.60 | 0.85 |
| DBP, mmHg | 77.7 ± 10.6 | 77.7 ± 10.30 | 0.97 |
| FPG, mmol/L | 5.91 ± 0.31 | 5.90 ± 0.29 | 0.57 |
| Cholesterol, mmol/L | 5.02 ± 0.87 | 4.99 ± 0.89 | 0.44 |
| Triglyceride, mmol/L | 1.77 ± 1.21 | 1.79 ± 1.37 | 0.77 |
| HDL, mmol/L | 1.38 ± 0.29 | 1.36 ± 0.29 | 0.17 |
| LDL, mmol/L | 2.88 ± 0.69 | 2.87 ± 0.71 | 0.85 |
| ALT, U/L | 28.3 ± 20.8 | 28.9 ± 24.40 | 0.60 |
| AST, U/L | 26.90 ± 9.60 | 27.30 ± 12.20 | 0.49 |
| BUN, mmol/L | 5.02 ± 1.22 | 5.01 ± 1.19 | 0.77 |
| CCR, μmol/L | 75.00 ± 14.70 | 74.80 ± 14.90 | 0.87 |

Data are shown as means ± standard deviation for normally distributed variables and percentages for categorical variables. BMI, body mass index; SBP, systolic blood pressure; DBP, diastolic blood pressure; FPG, fasting plasma glucose; HDL, high - density lipoprotein; LDL, low - density lipoprotein; ALT, alanine aminotransferase; AST, aspartate aminotransferase; BUN, blood urea nitrogen; CCR, creatinine clearance rate.
