# Supplementary material for: A predictive study of glycaemic reversal in Chinese individuals with prediabetes based on machine learning: a 5-year cohort study
Source: Front Endocrinol (Lausanne). 2026 Jan 28;17:1686082. doi: 10.3389/fendo.2026.1686082 (PMC12890694; doi:10.3389/fendo.2026.1686082)
Supplement: Supplementary file 3 [file Table3.docx]

**S Table 3. Comparison of clinical indicators between participants with prediabetes and those with normoglycemia in the training set.**

| Variables | Prediabetes  (n =610) | Normoglycemia  (n = 646) | P value |
| --- | --- | --- | --- |
| Male, n (%) | 463 (75.90 %) | 462 (71.50 %) | 0.09 |
| Smoking history, n (%) | 197 (32.30 %) | 168 (26.00 %) | 0.02 |
| Drinking history, n (%) | 159 (26.10 %) | 187 (28.90 %) | 0.28 |
| Family history of diabetes, n (%) | 45 (7.40 %) | 37 (5.70 %) | 0.29 |
| Age, years | 50.41 ± 11.39 | 43.43 ± 11.14 | <0.01 |
| BMI, kg/m^2^ | 25.17 ± 2.97 | 24.26 ± 3.31 | <0.01 |
| SBP, mmHg | 126.25 ± 16.29 | 121.59 ± 14.67 | <0.01 |
| DBP, mmHg | 79.25 ± 10.29 | 76.27 ± 10.14 | <0.01 |
| FPG, mmol/L | 5.99 ± 0.32 | 5.82 ± 0.23 | <0.01 |
| Cholesterol, mmol/L | 5.07 ± 0.88 | 4.91 ± 0.88 | <0.01 |
| Triglyceride, mmol/L | 1.90 ± 1.36 | 1.68 ± 1.38 | <0.01 |
| HDL, mmol/L | 1.36 ± 0.30 | 1.36 ± 0.28 | 0.92 |
| LDL, mmol/L | 2.93 ± 0.71 | 2.81 ± 0.70 | <0.01 |
| ALT, U/L | 30.59 ± 29.08 | 27.24 ± 18.71 | 0.02 |
| AST, U/L | 28.05 ± 14.16 | 26.51 ± 10.05 | 0.03 |
| BUN, mmol/L | 5.11 ± 1.19 | 4.91 ± 1.18 | <0.01 |
| CCR, μmol/L | 74.64 ± 14.80 | 75.00 ± 15.04 | 0.67 |

Data are shown as means ± standard deviation for normally distributed variables and percentages for categorical variables. BMI, body mass index; SBP, systolic blood pressure; DBP, diastolic blood pressure; FPG, fasting plasma glucose; HDL, high - density lipoprotein; LDL, low - density lipoprotein; ALT, alanine aminotransferase; AST, aspartate aminotransferase; BUN, blood urea nitrogen; CCR, creatinine clearance rate.
