# Supplementary material for: A predictive study of glycaemic reversal in Chinese individuals with prediabetes based on machine learning: a 5-year cohort study
Source: Front Endocrinol (Lausanne). 2026 Jan 28;17:1686082. doi: 10.3389/fendo.2026.1686082 (PMC12890694; doi:10.3389/fendo.2026.1686082)
Supplement: Supplementary file 4 [file Table4.docx]

**S Table 4. Coefficient of variable selection from LASSO regression.**

| Variables | Coefficient |
| --- | --- |
| Age, years | -0.025513183 |
| BMI, kg/m2 | -0.025639660 |
| SBP, mmHg | -0.001049344 |
| DBP, mmHg | -0.002779086 |
| FPG, mmol/L | -0.938063895 |
| Cholesterol, mmol/L | 0.184032520 |
| Triglyceride, mmol/L | -0.049856610 |
| LDL, mmol/L | -0.348593693 |
| ALT, U/L | -0.005720750 |
| AST, U/L | 0.005964856 |
| BUN, mmol/L | 0.036296241 |
| Smoking history, n | -0.254540994 |

BMI, body mass index; SBP, systolic blood pressure; DBP, diastolic blood pressure; FPG, fasting plasma glucose; LDL, low - density lipoprotein; ALT, alanine aminotransferase; AST, aspartate aminotransferase; BUN, blood urea nitrogen; LASSO, least absolute shrinkage and selection operator.
